# Heightened Local T_h_17 Cell Inflammation Is Associated with Severe Community-Acquired Pneumonia in Children under the Age of 1 Year

**DOI:** 10.1155/2021/9955168

**Published:** 2021-09-22

**Authors:** Ming Liu, Bingtai Lu, Huifeng Fan, Xuanjie Guo, Shuling Du, Diyuan Yang, Yiping Xu, Yue Li, Di Che, Yunfeng Liu, Xiaoqiong Gu, Tao Ding, Ping Wang, Hai-bin Luo, Gen Lu

**Affiliations:** ^1^Department of Respiratory, Guangzhou Institute of Pediatrics, Guangzhou Women and Children's Medical Centre, Guangzhou Medical University, Guangzhou, Guangdong, China; ^2^School of Pharmaceutical Sciences, Sun Yat-sen University, Guangzhou, Guangdong, China; ^3^Medical Research Center of Guangdong Provincial People's Hospital and Guangdong Academy of Medical Sciences, Guangzhou, Guangdong, China; ^4^School of Life Sciences, Sun Yat-sen University, Guangzhou, Guangdong, China; ^5^Department of Immunology, Zhongshan School of Medicine, Sun Yat-sen University, Guangzhou, Guangdong, China; ^6^Department of Neonatology, Guangzhou Institute of Pediatrics, Guangzhou Women and Children's Medical Centre, Guangzhou Medical University, Guangzhou, Guangdong, China

## Abstract

Severe community-acquired pneumonia (sCAP) early in life is a leading cause of morbidity, mortality, and irreversible sequelae. Herein, we report the clinical, etiological, and immunological characteristics of 62 children age < 1 year. We measured 27 cytokines in plasma and bronchoalveolar lavage (BAL) from 62 children age < 1 year who were diagnosed with CAP, and then, we analyzed correlations among disease severity, clinical parameters, and etiology. Of the entire cohort, three cytokines associated with interleukin-17- (IL-17-) producing helper T cells (T_h_17 cells), IL-1*β*, IL-6, and IL-17, were significantly elevated in sCAP patients with high fold changes (FCs); in BAL, these cytokines were intercorrelated and associated with blood neutrophil counts, Hb levels, and mixed bacterial-viral infections. BAL IL-1*β* (area under the curve (AUC) 0.820), BAL IL-17 (AUC 0.779), and plasma IL-6 (AUC 0.778) had remarkable predictive power for sCAP. Our findings revealed that increased local T_h_17 cell immunity played a critical role in the development of sCAP in children age < 1 year. T_h_17 cell-related cytokines could serve as local and systemic inflammatory indicators of sCAP in this age group.

## 1. Introduction

Community-acquired pneumonia (CAP) is the leading cause of morbidity and mortality in children under 5 years of age [1–4]. Despite the advances made in antimicrobial therapy and critical care, children with severe CAP (sCAP) can experience multiple pulmonary complications, including empyema, lung abscess, pneumothorax, acute respiratory-distress syndrome (ARDS), and chronic respiratory failure, requiring tracheostomy and prolonged mechanical ventilation [5]. Long-term adverse sequelae, including restrictive and obstructive lung diseases, bronchiectasis, interstitial lung disease, and asthma, are also common among children hospitalized with sCAP [6, 7].

Young age, especially < 1 year, is an additional risk factor for sCAP [8]. A retrospective multicenter cohort study that enrolled 16,162 children with pneumonia pointed out that at age 2-11 months (vs. 12-59 months), elevated respiratory rate, and weight-for-age *Z*-score were highly discriminative factors for mortality [9]. Furthermore, the first few months of life are important to promoting childhood respiratory health; lower-respiratory-tract infection early in life can impair lung function [10]. Consistent with the above findings, in our retrospective study involving 25,564 children ages 0–5 years diagnosed with CAP, most of the children were age < 1 year (59.0% of all patients), and this subgroup had the highest incidences of sCAP (70.5% of all severe cases) and mortality (76.6% of all mortalities; unpublished data). This prompted us to investigate the factors associated with sCAP in this age group.

Interleukin-17- (IL-17-) producing helper T cells (T_h_17 cells) play important roles in host defense against pneumonic and other invasive pathogens [11]. IL-17 is produced by activated T cells that are positive for clusters of differentiation 4 and 8 (CD4^+^ and CD8^+^ T cells), T cells, invariant natural-killer T (iNKT) cells, type 3 innate lymphoid cells (ILC3s), and mucosal-associated invariant T (MAIT) cells [12, 13]. IL-6 favors T_h_17 cell over regulatory T-cell (Treg) differentiation [14] by promoting activation of transcription 3 (STAT3) [15]. IL-1b is another T_h_17 cell–inducing cytokine that suppresses IL-10 production [16, 17]. T_h_17 cell responses are reported to be involved in the progression of certain diseases, including autoimmune diseases [18], chronic inflammatory diseases [19], and cancer [20]. However, the magnitude of these cytokines and their correlations with the etiology of pneumonia during infancy have scarcely been studied.

Neonates and infants display distinct differences in immune cell phenotypes and functions compared with older children and adults [21–23]. Currently, diagnosis of sCAP at a very young age largely relies on clinical symptoms [24]. Although there were studies showing that cytokines correlated to childhood pneumonia severity [25–27], most of the studies were conducted from serum. Moreover, factors associated with sCAP are rarely studied in patients age < 1 year. Herein, we report the clinical and etiological characteristics of and cytokine profiles from blood and BAL taken from 62 children age < 1 year. We also analyzed factors associated with disease severity and the connection between infectious pathogens and immune responses. This study could provide insights into early prediction of severe pneumonia in infancy.

## 2. Methods

### 2.1. Ethics Statement

All of the procedures in this study involving human participants were performed in accordance with the ethical standards of the institutional and/or national research committee, as well as with the 1964 Declaration of Helsinki and its later amendments or with comparable ethical standards. For the 62 children enrolled in the cytokine studies, we obtained signed written consent from their parents or other legal guardians. This study was approved by the Medical Ethics Committee of Guangzhou Women and Children's Medical Center (GWCMC; Guangzhou, China; Approval No. 2016111853).

### 2.2. Study Design and Cohort Assembly

From January 2017 to January 2020, we evaluated 743 patients age < 1 year who were diagnosed with CAP at GWCMC. Exclusion criteria were as follows: (1) known or suspected active tuberculosis, (2) severe concomitant disease (chronic pulmonary disease, severe cardiovascular disease, neoplasia, or kidney or liver disease), (3) primary immunodeficiency, (4) acquired immunodeficiency syndrome (AIDS) and immunosuppressive medications taken before admission, and (5) lack of eligible data or of paired blood/bronchoalveolar-lavage (BAL) samples. Cases were diagnosed as severe according to the following criteria, as previously described [24, 28]: (1) Major criteria: (a) invasive mechanical ventilation; (b) fluid refractory shock; (c) acute need for noninvasive positive-pressure ventilation; and (d) hypoxemia requiring fraction of inspired oxygen (FiO2) > inspired concentration or flow feasible in the general-care area. (2) Minor criteria: (a) respiratory rate > World Health Organization (WHO) classification for the patient's age; (b) apnea; (c) increased work of breathing (e.g., retractions, dyspnea, nasal flaring and grunting); (d) arterial-oxygen partial pressure (PaO2)/FiO2 ratio < 250; (a) multilobar infiltrates; (e) Pediatric Early Warning Score > 6; (f) altered mental status; (g) hypotension; (h) presence of effusion; (i) comorbidities; and (j) unexplained metabolic acidosis.

### 2.3. Clinical Data Collection

We retrieved information on demographic characteristics, disease severity, and pathogens from each patient's electronic medical record (EMR). In the group consisting of children age < 1 year, 21 cases were diagnosed with severe pneumonia, the rest (*n* = 41) with nonsevere pneumonia. From each patient, we collected blood, induced sputum, and lung aspirate, in which we tested 18 common types of pathogens known to cause pneumonia using standard laboratory tests: respiratory syncytial virus (RSV), human adenovirus (HAdV), human parainfluenza virus (HPIV), *Rhinovirus* (RHV), *Cytomegalovirus* (CMV), influenza virus (IFV), human bokavirus (HBoV), *human metapneumovirus* (HMPV), *enterovirus* (EV), *Mycoplasma pneumoniae* (MP), *Haemophilus influenzae* (HI), *Staphylococcus aureus* (SA), *Streptococcus pneumoniae* (SP), *Pseudomonas aeruginosa* (PA), *Klebsiella pneumoniae* (KP), *Moraxella catarrhalis* (MC), *Baumannii* (BM), and *Stenococcus maltophilia* (SM).

### 2.4. Bronchoalveolar Lavage and Plasma Sample Collection

We collected all plasma and BAL specimens during the patient's acute phase before corticosteroid treatment was administered. All the collected BAL samples here were SARS-CoV-2 negative. BAL samples were collected from children with CAP via flexible fiberoptic bronchoscopy. The surgery was performed in the Respiratory Department of GWCMC, focusing on the most radiologically and/or endoscopically affected areas. We injected warm sterile saline (2–3 ml/kg body weight) into each affected site and recovered it via aspiration into a suction trap as a BAL sample. Plasma samples were collected from remaining venous blood after clinical examinations. Both types of samples were processed concomitantly and stored in 4°C for subsequent cytokine assays.

### 2.5. Cytokine Assays

We used a Bio-Plex Pro Human Cytokine Standard 27-Plex magnetic-bead–based multiplex immunoassay with a Group I-kit on a Luminex Bio-Plex 200 system (all from BioRad Laboratories, Hercules, CA, USA) per manufacturer's instructions in order to assay cytokines in plasma and BAL samples. The kit included the following cytokines: IL-1*β*; IL-1RA; IL-2; IL-4; IL-5; IL-6; IL-7; IL-8; IL-9; IL-10; IL-12 (p70); IL-13; IL-15; IL-17A; basic fibroblast growth factor (bFGF); eotaxin; granulocyte colony-stimulating factor (G-CSF); granulocyte-macrophage colony-stimulating factor (GM-CSF); interferon gamma (IFN-*γ*); interferon gamma inducible protein 10 kD (IP-10); monocyte chemoattractant protein 1 (MCP-1); macrophage inflammatory proteins 1*α* and 1*β* (MIP-1*α*, MIP-1*β*); regulated on activation, normal T cell expressed, and secreted (RANTES); tumor necrosis factor alpha (TNF-*α*); platelet-derived growth factor with two B subunits (PDGF-BB); and vascular endothelial growth factor (VEGF). If results were under the limits of detection, we used the lowest detection threshold for statistical analysis.

### 2.6. Statistics

All of the data were analyzed using GraphPad Prism version 7.0 (GraphPad Software, Inc., San Diego, CA, USA) or SPSS version 25 (IBM Corp., Armonk, NY, USA). We calculated *P* values using the Wilcoxon matched-pair signed-rank test for paired samples, the Mann–Whitney *U* test for unpaired samples, and Kruskal–Wallis test for multiple comparison. Correlations were determined using Spearman's linear regression. *P* values for contingency were determined by Fisher's exact test. Receiver operating characteristic (ROC) curves were constructed in SPSS to evaluate the diagnostic accuracy of differences in cytokine levels between sCAP and nonsevere CAP (nsCAP).

## 3. Results

### 3.1. Clinical Characteristics of Studied Cohort

**Figure 1:**
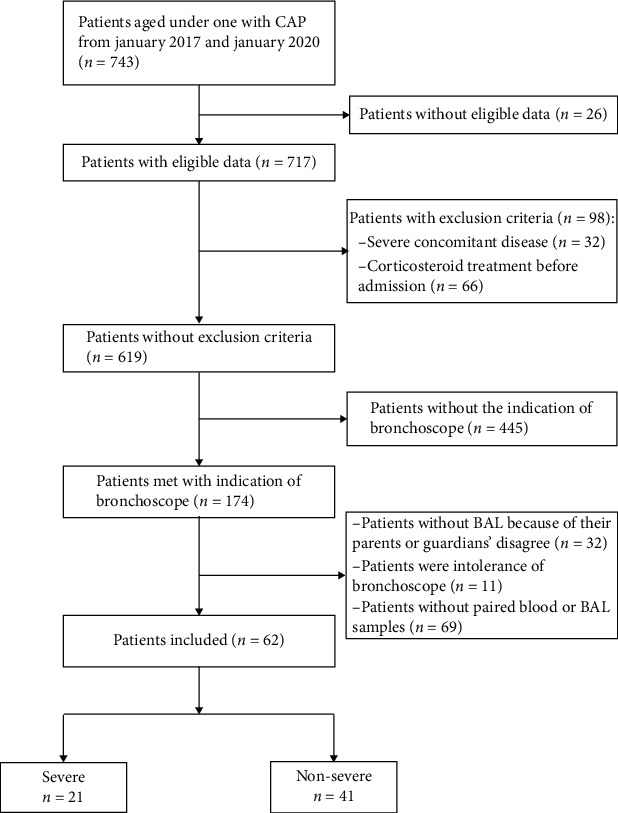
Flow chart and inclusion criteria of the study design.

Patients' baseline demographic and clinical characteristics and comparisons between subgroups (sCAP vs. nsCAP) are presented in Table 1 and Figure 1. Overall, median (interquartile range (IQR)) age was 8 (range, 5-10) months. Forty-six (74.2%) patients were male. Signs and symptoms included fever (45.2%), cough (91.9%), wheezing (50.0%), dyspnea (33.9%), change in level of consciousness (12.9%), and digestive symptoms (16.1%). Single-viral, bacterial, and mixed bacterial-viral infections were detected in plasma and BAL from, respectively, 19 (30.6%), 13 (21.0%), and 12 (19.4%) of the total 62 cases. Antibiotics and systemic corticosteroids were administered in 35 (56.5%) and 6 (9.7%) cases during whole medication, respectively. Median length of hospital stay was 9 (range, 4-39) days.

Twenty-one (33.9%) patients were diagnosed with sCAP. Patients in the severe (S) group presented with significantly more fever, wheezing, dyspnea, loss of consciousness, and digestive symptoms (*P* < 0.05) than those in the nonsevere (NS) group (Table 1). Moreover, compared with the NS group, the S group had significantly decreased levels of blood hemoglobin (Hb; S vs. NS, 101 (range, 92–110) vs. 118 (range, 107–125) × 10^9^/l; *P* < 0.0001) and platelet counts (S vs. NS, 335 (range, 279–419.5) vs. 403 (range, 318–503) × 10^9^/l; *P* = 0.0261) and elevated levels of lactate dehydrogenase (LDH; S vs. NS, 369.5 (range, 336–647.5) vs. 309 (range, 293–356) U/l; *P* = 0.0224). Notably, a significantly higher percentage of patients in the NS group had single-bacterial infections (S vs. NS, 4.8% vs. 28.6%; *P* = 0.0445). In contrast, patients in the S group had significantly more mixed bacterial-viral infections (S vs. NS, 42.8% vs. 7.1%; *P* = 0.0016). Children in the S group had longer hospital stays and were more likely to be administered antibiotics and corticosteroids (*P* < 0.05). 31 patients, including all severe patients, were given antibiotics 1 or 2 days before flexible fiberoptic bronchoscopy. Patients who had received antibiotic treatments had significantly higher cytokine profile than those who did not receive antibiotics (data not showed). No mortalities were reported in this cohort.

### 3.2. Inflammatory Cytokines Were Significantly Elevated in BAL

**Figure 2:**
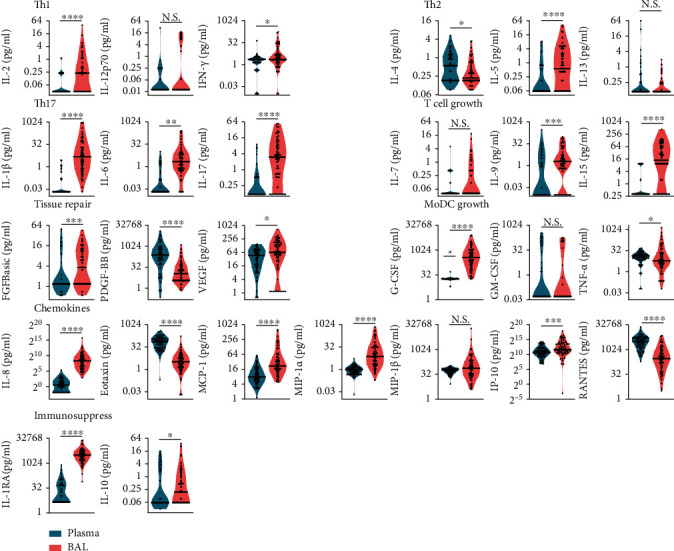
Inflammatory cytokine levels in plasma and BAL from children age < 1 year (plasma, *n* = 62; BAL, *n* = 62). Cytokine levels are presented as box plots outlined with kernel probability density. Medians and quartiles are indicated with whiskers reaching up to 1.5× IQR. The width of the colored area represents the proportion of data (green, plasma; red, BAL). Levels of plasma and BAL cytokines were compared in pairs using the Wilcoxon test; ^∗^*P* < 0.05, ^∗∗^*P* < 0.01, ^∗∗∗^*P* < 0.001, ^∗∗∗∗^*P* < 0.0001.

**Figure 3:**
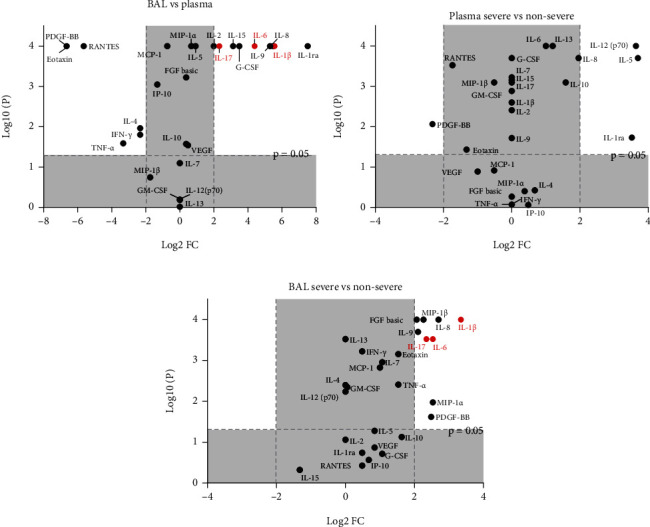
Volcano plots showing significance and fold changes of inflammatory cytokines. The *x*-axis shows log^2^ FCs in median BAL vs. plasma cytokine levels (a), median cytokine levels in plasma in the severe (S) vs. nonsevere (NS) group (b), and median cytokine levels in BAL in the S vs. NS group (c). The *y*-axis shows the log^10^*P* value of cytokine levels being statistically compared. *P* values were calculated using Wilcoxon's (a) or the Mann–Whitney *U* (b, c) test; ^∗^*P* < 0.05, ^∗∗^*P* < 0.01, ^∗∗∗^*P* < 0.001, ^∗∗∗∗^*P* < 0.0001. Grey area represents no significant change (*P* > 0.05) or within 2 log FC. T_h_17 cell-related cytokines are highlighted in red.

We compared levels of 27 inflammatory cytokines between paired BAL and plasma samples. Sixteen cytokines were statistically significantly upregulated in BAL versus plasma (Figure 2). Median concentrations of IL-1*β*, IL-6, and IL-17 (T_h_17 cell polarization); IL-9 and IL-15 (T-cell differentiation); G-CSF (myeloid-cell stimulation); IL-1RA (immunosuppression); and IL-8 (chemokines) were significantly increased in BAL, by > 2 logs of fold change (logFC; Figures 2 and 3(a)). Conversely, levels of IFN-g (T_h_1 cell polarization), IL-4 (T_h_2 cell polarization), PDGF-BB (tissue repair), eotaxin and RANTES (chemokines), and TNF-*α* (myeloid-cell stimulation) were significantly downregulated in BAL (by > 2 logFC; Figures 2 and 3(a)).

### 3.3. T_h_17 Cell-Related Cytokines Were Significantly Elevated in the Severe Group

**Figure 4:**
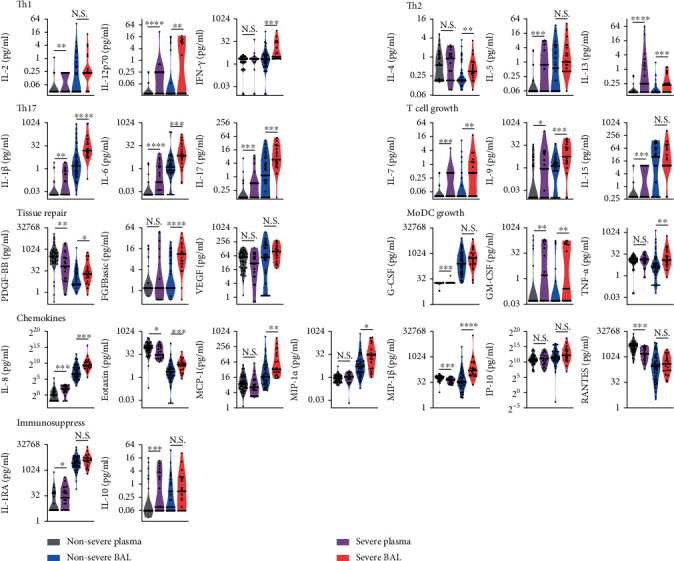
Inflammatory-cytokine levels in plasma and BAL from children < 1 year, grouped by disease severity (S, *n* = 21; NS, *n* = 41). Cytokine levels are presented as box plots outlined with kernel probability density. Medians and quartiles are indicated with whiskers reaching up to 1.5× IQR. The width of the colored area represents the proportion of data (grey, NS plasma; purple, S plasma; blue, NS BAL; red, S BAL). *P* values were calculated using the Mann–Whitney *U* test; ^∗^*P* < 0.05, ^∗∗^*P* < 0.01, ^∗∗∗^*P* < 0.001, ^∗∗∗∗^*P* < 0.0001.

When comparing cytokine levels in plasma samples between the S and the NS groups, we found that three cytokines—IL-1RA (immunosuppression), IL-5 (T_h_2 cell polarization), and IL-12 (T_h_1 cell polarization; Figures 3(b) and 4) were significantly upregulated (by 2 logFC). Conversely, 10 cytokines—IL-1*β*, IL-6, and IL-17 (T_h_17 cell polarization); MIP-1*α*, MIP-1*β*, and IL-8 (chemokines); PDGF-BB and bFGF (tissue repair); and IL-9 (T-cell survival)—in BAL were significantly elevated (by 2 logFC; Figures 3(c) and 4).

### 3.4. T_h_17-Related Cytokines Were Associated with Blood Neutrophil Count, Hemoglobin Level, and Mixed Bacterial-Viral Infections

**Figure 5:**
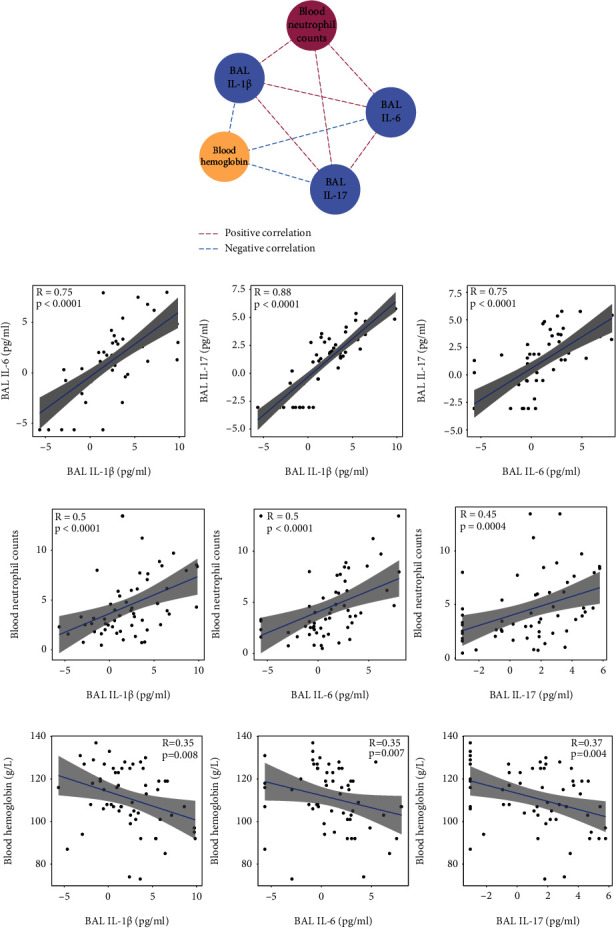
Clinical associations of T_h_17 cell–associated cytokines. (a) Significant correlations of BAL T_h_17 cell–associated cytokines (IL-1b, IL-6, and IL-17, shown in blue), blood neutrophil counts (red), and Hb (yellow). Positive correlations are shown by red dashed lines, while negative correlations are shown by blue dashed lines. Significant correlations were defined as *P* < 0.05. (b) T_h_17 cell-related cytokines (BAL IL-1b, IL-6, and IL-17) were positively intercorrelated. (c, d) T_h_17 cell-related cytokines (BAL IL-1b, IL-6, and IL-17) were correlated positively with blood neutrophil counts (c) and negatively with Hb (d). Blue lines represent linear-regression models. Grey area represents the confidence interval of the model. Statistics are presented in the models.

We further investigated the association between T_h_17 cell cytokines and the clinical parameters of the cohort. Strong intercorrelations were found between concentrations of IL-1b and those of IL-17, IL-6, and IL-17 in BAL. Moreover, BAL IL-1b, IL-6, and IL-17 were correlated positively with blood neutrophil count and negatively with Hb (Figures 5(a) and 5(b)). No correlation was found between plasma T_h_17 cell-related cytokines and patients' clinical parameters.

**Figure 6:**
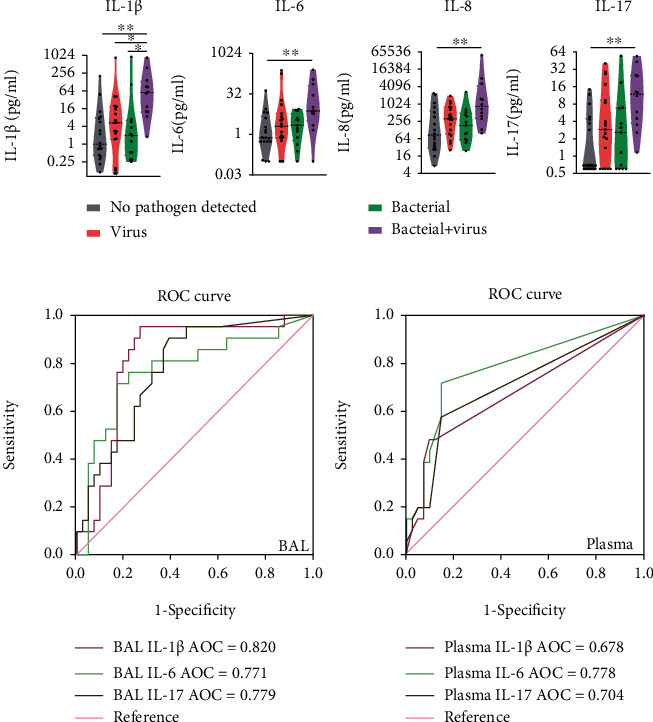
T_h_17 cell–associated cytokines were elevated in mixed bacterial-viral infections and were good predictors for sCAP. (a) Comparisons of BAL IL-1*β*, IL-6, and IL-17 levels of children with CAP in whom neither viruses nor bacteria were detected (*n* = 18), in whom only viruses were detected (*n* = 19), in whom only bacteria were detected (*n* = 13), and in whom mixed bacterial-viral infections were detected (*n* = 12). Cytokine levels are presented as box plots outlined with kernel probability density. Medians and quartiles are indicated with whiskers reaching up to 1.5× IQR. The width of the colored area represents the proportion of data (grey, no viruses or bacteria detected; red, viruses only; green, bacteria only; purple, mixed bacterial-viral infections). *P* values were calculated using the Kruskal–Wallis test with correction for multiple comparisons using statistical hypothesis testing (Dunn's test). (b) ROC curves for BAL IL-1*β*, IL-6, and IL-17 for discriminating sCAP from nsCAP.

To explore the correlation between infectious etiology and cytokine responses, we examined the associations between the cytokine profiles of CAP patients and viral or bacterial infections. IL-1*β* was significantly upregulated in BAL from children with mixed bacterial-viral infections compared with those infected by either bacterial or viral pathogens alone (Figure 6(a)). It should be noted that 11 out of 12 patients with mixed bacterial-viral infections were infected with adenovirus. In addition, BAL IL-6 and IL-17 also trended toward upregulation in patients with mixed bacterial-viral infections (Figure 6(a)). We observed no changes in T_h_17 cell-related cytokine levels in BAL between single-viral and single-bacterial infections. No alterations in serum cytokine levels were associated with mixed bacterial-viral infections (data not shown).

We further investigated the correlations between T_h_17 cell-related cytokines and individual pathogens. As shown in Table S1, patients in the severe group had significantly higher percentages of respiratory syncytial virus (RSV), human adenovirus (HAdV), *Pseudomonas aeruginosa* (PA), and *Baumanii* (BM) infections. Patients with HAdV infections were positively correlated with all three T_h_17 cell-related cytokines. Moreover, for RSV and BM infections, at least one T_h_17 cell-related cytokine was elevated.

### 3.5. T_h_17 Cell-Related Cytokines Could Be Potential Predictors of sCAP

To evaluate the diagnostic value of these cytokines for CAP severity, we performed standard receiver operating characteristic (ROC) curve analyses. BAL IL-1*β* (area under the curve (AUC) 0.820), BAL IL-17 (AUC 0.779), and plasma IL-6 (AUC 0.778) had good predictive value for sCAP. BAL IL-1*β* was the best analyte by which to discriminate between sCAP and nsCAP in children (Figure 6(b)). The optimal cut-off points for these cytokines were as follows: BAL IL-1*β*, ≥ 5.355 pg/ml; BAL IL-17, ≥ 2.52 pg/ml; and plasma IL-6, ≥ 0.266 pg/ml. Combinations of cytokines from BAL or plasma also had good predictive effect for sCAP but were not better than individual cytokines (Fig. S1, Table 2).

## 4. Discussion

In this study, we investigated clinical characteristics of and inflammatory cytokines in plasma and BALs from a cohort of 62 pneumonia patients age < 1 year. We found that levels of multiple inflammatory cytokines, including T_h_17 cell-related cytokines (IL-1b, IL-6, and IL-17), were significantly elevated in BAL versus plasma. Moreover, concentrations of T_h_17 cell-related cytokines in BAL were even more significantly upregulated, by > 2-logs, in the S group versus the NS group. T_h_17 cell-related cytokines were intercorrelated, positively correlated with blood neutrophil count, and negatively correlated with Hb levels. Furthermore, elevated T_h_17 cell response was associated with mixed bacterial-viral infections. In addition, we demonstrated that T_h_17 cell-related cytokines from both plasma and BAL served as good predictors of sCAP in patients age < 1 year.

T_h_17 cell responses to pneumonia have been extensively studied in a mouse model [29] as well as in humans [30]. Herein, we are the first to describe T_h_17 cell involvement as a major feature of sCAP in very young patients. The role of IL-17 in the pathogenesis of pneumonia remains controversial. Although it is important in host defense against infections [29], it has been identified as pathological in both acute and chronic pulmonary inflammation [31, 32]. When IL-17 binds to its receptor IL-17R, which is mainly expressed on epithelial cells (ECs), endothelial cells, fibroblasts, macrophages, and dendritic cells (DCs), downstream nuclear factor *κ*-light-chain-enhancer of activated B cells (NF-*κ*B) signaling is activated, inducing production of G-CSF and IL-8 by macrophages and ECs, which subsequently promotes neutrophil infiltration [33–35]. Consistent with these findings, we showed that T_h_17 cell-related cytokines were positively correlated with blood neutrophil count. Although we did not directly investigate the correlation between IL-17 and pulmonary neutrophils, the increase in blood neutrophil counts and percentages implied that neutrophils had migrated from bone marrow to the site of inflammation. This correlation suggested that pulmonary T_h_17 cell-related cytokines could promote lung inflammation by excessive neutrophil recruitment. Our previous study showed that pulmonary IL-17 was mainly produced by CD4^+^ T (T_h_17) and MAIT (MAIT17) cells in the lungs of CAP patients [36]; those cells were locally induced by the inflammatory pulmonary environment to produce IL-17. Blockade of IL-1*β* and IL-6 partially attenuated IL-17 production, suggesting that IL-1*β* and IL-6 supported the differentiation of T_h_17 and MAIT17 cells. In accordance with our previous study, we found that the level of BAL IL-17 was positively correlated with IL-1*β* and IL-6 and that the correlation was highly significant. Taking these findings as a whole, we reasoned that pulmonary IL-17 was produced due to the induction of local inflammatory cytokines (IL-1*β* and IL-6).

Bacterial infections are reported to be associated with CAP severity in children [37, 38]. However, these studies do not illustrate the effects of mixed infections on disease severity in pediatric CAP. In this study, we showed that children age < 1 year with sCAP had a significantly increased proportion of mixed bacterial-viral infections. This was consistent with previous studies in adult CAP patients showing that mixed bacterial-viral infections were related to disease severity, mortality, and complicated course [39–41]. Notably, 11 out of 12 patients with mixed bacterial-viral infections were human adenovirus positive. Similar to the previous findings that adenovirus coinfections aggravated mycoplasma pneumonia severity [42], our results suggested that adenovirus-bacterial coinfections aggravated pneumonia severity of children under year 1. Viral and bacterial pathogens can both elicit host T_h_17 cell response. For instance, T_h_17 cell effector cytokines (IL-1*β*, IL-17A, and IL-22) are upregulated in the plasma of patients with laboratory-confirmed A (H3N2) influenza and/or HPIV infections [43]. In a retrospective multiple-center study that enrolled 267 laboratory-confirmed coronavirus disease 2019 (COVID-19) cases, most patients had high plasma concentrations of IL-6 and IL-17A [44]. Infection with SP, a major bacterial cause of CAP, promoted pulmonary IL-17 production in a murine model [11]. Herein, we showed that BAL T_h_17 cell-related cytokines, especially IL-1*β*, were further upregulated in patients with mixed bacterial-viral infections than in those with single-type infections. We did not find a correlation between plasma cytokine levels and pathogen species. This supported the theory that the immune response in the lung, but not the systemic immune response, was more likely influenced by the types of invading pathogens.

In our study, 33.9% of hospitalized patients developed sCAP; these patients had higher neutrophil counts and LDH levels and lower Hb levels and platelet counts. However, levels of C-reactive protein (CRP) and procalcitonin (PCT), which have long been used as inflammatory indicators in disease diagnosis, did not differ significantly between the S and the NS groups. In the search for potential biomarkers for the prediction of sCAP, we found that BAL IL-1*β* (AUC 0.820), BAL IL-17 (AUC 0.779), and plasma IL-6 (AUC 0.778) could efficiently discriminate sCAP from nsCAP. Since plasma is easier to obtain than BAL, plasma IL-6 is the best indicator of the three. IL-6 is considered as a biomarker associated with pneumonia diagnosis and with bacterial infection and outcome in adult and pediatric CAP patients [45, 46]. In this study, we proved that it is also an effective biomarker in infant patients.

There were two major limitations of our study. First, the small sample size of this cohort restricted our power to include all types of clinical infections, especially mixed types. Future work must focus on investigating the detailed variations of cytokine profiles in pneumonia caused by different infection types. Second, because we could not obtain plasma and BAL samples from healthy children age < 1 year, this study lacked a healthy control group. Nevertheless, we gained solid conclusions that can help clinicians distinguish between sCAP and nsCAP features in this age group.

Overall, we demonstrated that increased local T_h_17 cell immunity played an indispensable role in the development of sCAP. It was not only associated with disease severity and clinical parameters but also linked with mixed bacterial-viral infections. This study will expand current knowledge of sCAP in children < 1 year, which could assist in establishing age-specific management of and therapy for CAP.

## Figures and Tables

**Table 1:** Demographic and clinical characteristics of collected patients, median (interquartile range, 25%-75%).

Parameters	All (*n* = 62)	Severe (*n* = 21, 33.9%)	Nonsevere (*n* = 41, 66.1%)	*P* value
Demographics				
Age (month)	8 (5-10)	8 (5-10)	7.5 (5-10)	0.7666^a^
Sex (M), (%)	46 (74.2%)	15 (71.4%)	31 (75.6%)	0.7648^a^
Sign and symptoms				
Fever, No. (%)	28 (45.2%)	16 (76.2%)	12 (29.3%)	0.0010^a^
Cough, No. (%)	57 (91.9%)	20 (95.2%)	37 (90.2%)	0.6541^a^
Wheezing, No. (%)	31 (50.0%)	18 (85.7%)	13 (31.7%)	0.0001^a^
Dyspnea, No. (%)	21 (33.9%)	17 (81.0%)	4 (9.8%)	<0.0001^a^
Change in level of consciousness, No. (%)	8 (12.9%)	8 (38.1%)	0 (0.0%)	<0.0001^a^
Digestive symptoms, No. (%)	10 (16.1%)	7 (33.3%)	3 (7.3%)	0.0236^a^
Laboratory findings^c^				
Leukocytes (× 10^9^/l; normal range 5–12)	10.7 (8.4-14.3)	9.6 (7.5-14.9)	11.2 (9.4-13.6)	0.4525^a^
Neutrophils (× 10^9^/l; normal range 2.0-7.2)	3.6 (2.3-6.1)	5.4 (3.5-7.9)	3.1 (2.0-4.6)	0.0347^a^
Hemoglobin (g/l; normal range 105-145)	113 (103-123)	101 (92-110)	118 (107-125)	<0.0001^a^
Platelets (× 10^9^/l; normal range 140–440)	390 (310-463)	335 (279-419.5)	403 (318-503)	0.0261^a^
ALT (U/l; normal range 9–50)	22 (18.5-35)	21.5 (19-33.5)	27 (13-49)	0.6401^a^
LDH (U/l; normal range 159–322)	359 (316-501)	369.5 (336-674.5)	309 (293-356)	0.0224^a^
PCT (ng/ml; normal range <0.1)	0.10 (0.05-0.22)	0.21 (0.04-1.33)	0.09 (0.04-0.18)	0.2106^a^
CRP (mg/l; normal range 0.0–6.0)	14.2 (1.3-36.1)	15.7 (1.7-36.2)	4.9 (1.8-34.4)	0.6344^a^
Etiology				
Single virus	19 (30.6%)	7 (33.3%)	12 (28.6%)	0.7767^b^
Single bacteria	13 (21.0%)	1 (4.8%)	12 (28.6%)	0.0445^b^
Mixed bacteria-virus	12 (19.4%)	9 (42.8%)	3 (7.1%)	0.0016^b^
Others/undetected	18 (29.0%)	4 (19.0%)	16 (38.1%)	0.1541^b^
Treatment				
Antibiotics, No. (%)	35 (56.5%)	17 (81.0%)	18 (43.9%)	0.0069^b^
Systemic corticosteroid, No. (%)	6 (9.7%)	6 (28.6%)	0 (0.0%)	0.0009^b^
Outcome				
Length of stay (days)	9 (4-39)	11 (8.5-18)	8 (6-10)	< 0.0001^b^
Mortality, No. (%)	0 (0.0%)	0 (0.0%)	0 (0.0%)	>0.9999^b^

^a^*P* value calculated by Mann-Whitney test. ^b^*P* value calculated by chi-square test. ^c^The data of laboratory findings were collected from patients with acute exacerbation.

**Table 2:** Diagnostic power of cytokine thresholds at best performance.

Predictors	Cut-point (pg/ml)	Sensitivity (%)	Specificity (%)	AUC (95% CI)
BAL IL-1*β*	5.355	95.2%	73.2%	0.820 (0.706–0.934)
BAL IL-6	4.345	71.4%	82.9%	0.771 (0.638–0.905)
BAL IL-17	2.52	90.5%	61.0%	0.779 (0.662–0.896)
BAL IL-1*β*+ IL-6+ IL-17	0.227	85.7%	58.5%	0.781 (0.662-0.899)
Plasma IL-1*β*	0.312	47.6%	90.2%	0.678 (0.555–0.800)
Plasma IL-6	0.266	76.2%	85.4%	0.778 (0.661–0.895)
Plasma IL-17	0.312	57.1%	85.4%	0.704 (0.581–0.828)
Plasma IL-1*β*+ IL-6+ IL-17	0.246	71.4%	82.9%	0.747 (0.613-0.881)

## Data Availability

The data used to support the findings of this study are available from the corresponding author upon request.

## Supplementary Materials

Supplementary 1 Figure S1: receiver operating characteristic curves for combinations of plasma and BAL IL-1*β*, IL-6, and IL-17 in order to discriminate sCAP from nsCAP. ROC curves for combination of IL-1*β*, IL-6, and IL-17 in plasma (left panel) or BAL (right panel) for discriminating sCAP from nsCAP.

Supplementary 2 Table S1: correlations of individual pathogens and T_h_17 cytokines. Associations of T_h_17 cytokines (IL-1b, IL-6, and IL-17) and individual pathogens.
