# Supplementary figures and images for: Heightened Local Th17 Cell Inflammation Is Associated with Severe Community-Acquired Pneumonia in Children under the Age of 1 Year
Source: Mediators Inflamm. 2021 Sep 22;2021:9955168. doi: 10.1155/2021/9955168 (PMC8482031; doi:10.1155/2021/9955168)

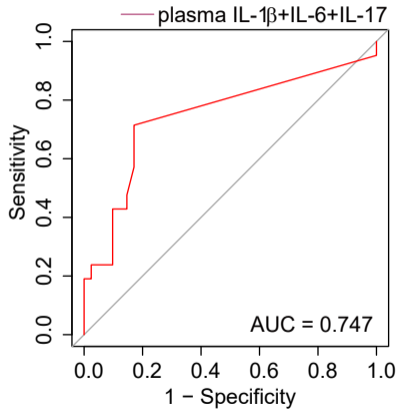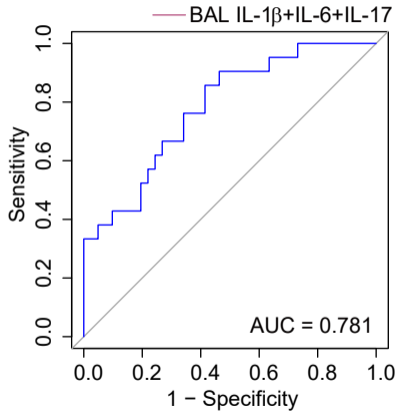

Supplement: Supplementary 1 — Figure S1: receiver operating characteristic curves for combinations of plasma and BAL IL-1β, IL-6, and IL-17 in order to discriminate sCAP from nsCAP. ROC curves for combination of IL-1β, IL-6, and IL-17 in plasma (left panel) or BAL (right panel) for discriminating sCAP from nsCAP. [file 9955168.f1.pdf]
