# Supplementary material for: Heightened Local Th17 Cell Inflammation Is Associated with Severe Community-Acquired Pneumonia in Children under the Age of 1 Year
Source: Mediators Inflamm. 2021 Sep 22;2021:9955168. doi: 10.1155/2021/9955168 (PMC8482031; doi:10.1155/2021/9955168)
Supplement: Supplementary 2 — Table S1: correlations of individual pathogens and Th17 cytokines. Associations of Th17 cytokines (IL-1b, IL-6, and IL-17) and individual pathogens. [file 9955168.f2.docx]

**Table S1 Correlations of individual pathogens and Th17 cytokines**

| Etiology | All (n=62) |  | Severe  (n=21, 33.9%) | Non-severe  (n=41, 66.1%) | *P-*value ^a^ |  | Elevated BAL Th17 cytokines  (vs others/undetected, *P*-value) ^b^ | | |
| --- | --- | --- | --- | --- | --- | --- | --- | --- | --- |
|  |  |  |  |  |  |  | IL-1β | IL-6 | IL-17 |
| Virus, No. (%) | 31(50.0%) |  | 16(76.2%) | 15(36.6%) | 0.003 |  |  |  |  |
| Respiratory syncytial virus (RSV) | 8(12.9%) |  | 5(23.8%) | 3(7.3%) | 0.007 |  | 0.03 | n.s. | 0.02 |
| Human adenovirus (HAdV) | 16(25.8%) |  | 10(47.6%) | 6(14.6%) | 0.005 |  | 0.002 | 0.005 | 0.002 |
| Human parainfluenza virus (HPIV) | 2(3.2%) |  | 1(4.8%) | 1(2.4%) | 0.6 |  | - | - | - |
| *Rhinovirus* (RHV) | 1(1.6%) |  | 0(0.0%) | 1(2.4%) | 0.5 |  | - | - | - |
| *Cytomegalovirus* (CMV) | 7(11.3%) |  | 2(9.5%) | 5(12.2%) | 0.8 |  | n.s. | n.s. | n.s. |
| Influenza virus (IFV) | 1(1.6%) |  | 1(4.8%) | 0(0.0%) | 0.2 |  | - | - | - |
| Human bokavirus (HBoV) | 0(0.0%) |  | 0(0.0%) | 0(0.0%) | - |  | - | - | - |
| Human *metapneumovirus* (HMPV) | 1(1.6%) |  | 0(0.0%) | 1(2.4%) | 0.5 |  | - | - | - |
| *Enterovirus* (EV) | 1(1.6%) |  | 1(4.8%) | 0(0.0%) | 0.2 |  | - | - | - |
| Bacterial No. (%) | 25(40.3%) |  | 10(47.6%) | 15(36.6%) | 0.4 |  |  |  |  |
| *Mycoplasma pneumoniae* (MP) | 8(12.9%) |  | 5(23.8%) | 3(7.3%) | 0.07 |  | 0.03 | n.s. | 0.02 |
| *Haemophilus influenzae* (HI) | 8(12.9%) |  | 4(19.0%) | 4(9.8%) | 0.3 |  | n.s. | 0.01 | 0.03 |
| *Staphylococcus aureus* (SA) | 11(17.7%) |  | 4(19.0%) | 7(17.1%) | 0.8 |  | n.s. | n.s. | n.s. |
| *Streptococcus pneumoniae* (SP) | 7(11.3%) |  | 3(14.3%) | 4(9.8%) | 0.6 |  | n.s. | n.s. | n.s. |
| *Pseudomonas aeruginosa* (PA) | 2(3.2%) |  | 2(9.5%) | 0(0.0%) | 0.04 |  | - | - | - |
| *Klebsiella pneumoniae* (KP) | 3(4.8%) |  | 1(4.8%) | 2(4.9%) | 1 |  | n.s. | n.s. | n.s. |
| *Moraxelle catarrhalis* (MC) | 3(4.8%) |  | 1(4.8%) | 2(4.9%) | 1 |  | n.s. | n.s. | n.s. |
| *Baumanii* (BM) | 3(4.8%) |  | 3(14.3%) | 0(0.0%) | 0.01 |  | 0.02 | n.s. | n.s. |
| *Stenococcus maltophilia* (SM) | 1(1.6%) |  | 1(4.8%) | 0(0.0%) | 0.2 |  | - | - | - |

^a^:*P* value calculated by Chi square’s test.

^b^: *P* value calculated by Mann-Whitney test.
